# Beneficial Effects of Bioactive Compounds in Mulberry Fruits against Cisplatin-Induced Nephrotoxicity

**DOI:** 10.3390/ijms19041117

**Published:** 2018-04-09

**Authors:** Dahae Lee, Jae Sik Yu, Seoung Rak Lee, Gwi Seo Hwang, Ki Sung Kang, Jae Gyu Park, Hyun Young Kim, Ki Hyun Kim, Noriko Yamabe

**Affiliations:** 1 School of Pharmacy, Sungkyunkwan University, Suwon 440-746, Korea; pjsldh@naver.com (D.L.); jsyu@bu.edu (J.S.Y.); davidseungrak@gmail.com (S.R.L.); 2 College of Korean Medicine, Gachon University, Seongnam 13120, Korea; seoul@gachon.ac.kr (G.S.H.); kkang@gachon.ac.kr (K.S.K.); 3 Advanced Bio Convergenve Center, Pohang Technopark, Pohang 37668, Korea; jaegpark@gmail.com; 4 Department of Food Science, Gyeongnam National University of Science and Technology, Jinju 52725, Korea; hykim@gntech.ac.kr

**Keywords:** mulberry, *Morus alba*, cisplatin, nephrotoxicity, mitogen-activated protein kinases, caspase-3

## Abstract

Mulberry, the fruit of white mulberry tree (*Morus alba* L., Moraceae), is commonly used in traditional Chinese medicines as a sedative, tonic, laxative, and emetic. In our continuing research of the bioactive metabolites from mulberry, chemical analysis of the fruits led to the isolation of five compounds, **1**–**5**. The compounds were identified as butyl pyroglutamate (**1**), quercetin 3-*O*-β-d-glucoside (**2**), kaempferol 3-*O*-β-d-rutinoside (**3**), rutin (**4**), and 2-phenylethyl d-rutinoside (**5**) by spectroscopic data analysis, comparing their nuclear magnetic resonance (NMR) data with those in published literature, and liquid chromatography–mass spectrometry analysis. The isolated compounds **1**–**5** were evaluated for their effects on anticancer drug-induced side effects by cell-based assays. Compound **1** exerted the highest protective effect against cisplatin-induced kidney cell damage. This effect was found to be mediated through the attenuation of phosphorylation of c-Jun N-terminal kinase, extracellular signal-regulated kinase, p38, mitogen-activated protein kinase, and caspase-3 in cisplatin-induced kidney cell damage.

## 1. Introduction

As the advancement in drug development increases, side effects are an important concern. Among these side effects, the most frequently studied factor is nephrotoxicity [1,2]. About 5% of the filtered drugs are reabsorbed actively and can reach high concentrations in the proximal tubular cells of the kidney [3,4]. In this condition, the kidneys do not function properly, affecting homeostasis [5,6].

Nephrotoxicity is induced by various drugs, such as acetaminophen (drug used to treat pain and fever), adriamycin, cisplatin, doxorubicin (anticancer drug), aminoglycosides, fluoroquinolones (antibiotics), and cyclosporine A (immunosuppressant) [7,8,9]. Among these drugs, cisplatin, a platinum-based anticancer drug, is effective in the treatment of human cervical cancer at various stages [10,11,12,13]. However, it has serious side effects that limit its usefulness. In particular, nephrotoxicity remains a major concern for anticancer chemotherapy using cisplatin [14,15].

Mulberry (*Morus alba* L.) is a well-known medicinal plant (Moraceae) widely distributed in Asia, North America, South America, Europe, and Africa; mulberry trees have been cultivated in China, Korea, and Japan as their leaves are used to feed silkworms (*Bombyx mori* L.). Different parts of the mulberry tree, including its root bark, leaves, and fruits, have been used in traditional Chinese medicines. The mulberry tree has shown various pharmaceutical benefits against cancer, aging, neurological and cardiovascular diseases, inflammation, diabetes, and bacterial infections [16,17,18]. Interestingly, mulberry fruits are often dried, or made into wine, juice, and jam worldwide [16,17]. Studies on the pharmaceutical potential and bioactive ingredients of mulberry have been conducted [16]. Mulberry has a number of bioactive compounds such as polyphenols, flavonoids, anthocyanins, and carotenoids; in particular, mulberry fruit has been reported to have various bioactive constituents, such as alkaloids, vitamins, fats (mainly linoleic acid, palmitic acid, oleic acid), and minerals [19,20,21]. These compounds exhibit potential bioactivities including antioxidative, anti-inflammatory, antitumor, and antidiabetic effects, cardiovascular, hepatoprotective, neuroprotective, hypoglycemic, hypotensive, and diuretic activities [16,22].

In our study of bioactive compounds from Korean medicinal plants, we investigated the bioactive constituents in mulberries and recently reported heterocyclic compounds and their antiangiogenic activities [23]. In our continuing research on the investigation of bioactive metabolites from mulberry, phytochemical analysis of the mulberry fruits led to the isolation of five compounds. In the present study, we investigated the health benefits of the isolated compounds on cisplatin-induced side effects by cell-based assays. We tested all the compounds isolated from mulberry fruits for their protective effects on cisplatin-induced cytotoxicity in LLC-PK1 kidney cells and identified their mechanism of action.

## 2. Results and Discussion

### 2.1. Isolation and Structural Identification of Compounds

**Figure 1:**
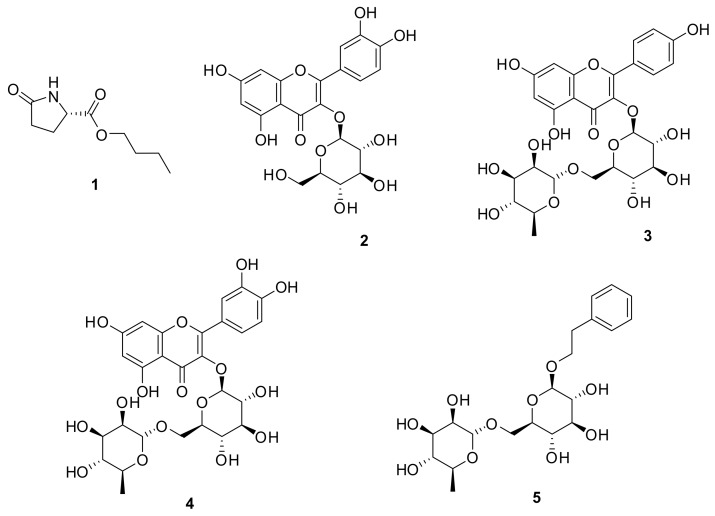
Chemical structures of compounds **1**–**5**.

The fruits of *M. alba* were extracted with 70% ethanol, and this ethanolic extract was sequentially separated by solvent partitioning (hexane, CH_2_Cl_2_, EtOAc, and *n*-BuOH) to obtain four main fractions. As the *n*-BuOH-soluble fraction accounted for a substantial amount of obtained fractions and the CH_2_Cl_2_-soluble fraction was already investigated in our previous study [23], phytochemical analysis was taken upon with the *n*-BuOH-soluble fraction in this study. Chemical analysis of the *n*-BuOH fraction led to the successful isolation of five compounds **1**–**5** (Figure 1) using column chromatography, preparative high-performance liquid chromatography (HPLC), and semi-preparative HPLC purification. The structures of the compounds were identified as butyl pyroglutamate (**1**) [24], quercetin 3-*O*-β-d-glucoside (**2**) [25], kaempferol 3-*O*-β-d-rutinoside (**3**) [26], rutin (**4**) [27], and 2-phenylethyl d-rutinoside (**5**) [28] by spectroscopic data analysis, comparing their nuclear magnetic resonance (NMR) data with those in published literature, and liquid chromatography–mass spectrometry (LC/MS) analysis.

Butyl pyroglutamate (**1**) could be an artifact during isolation because compound **1** was isolated from *n*-BuOH-soluble fraction. To identify whether compound **1** was a genuine natural compound or an isolation artifact, the crude ethanolic extract of *M. alba* fruits was analyzed by LC/MS and co-injected with the pure compound **1**. In the LC/MS analysis, we found a peak with the molecular ion corresponding to compound **1** in the crude ethanolic extract at the same retention time as that of compound **1**. This suggested that compound **1** was a genuine natural compound.

### 2.2. Bioactivities

Cisplatin is effective in the treatment of cervical cancer; however, its usefulness is often limited due to its severe side effects [10,29,30,31]. A major side effect in patients who have been prescribed anticancer drugs is nephrotoxicity. Cisplatin-induced nephrotoxicity is characterized by inflammation, apoptosis, or necrosis through accumulation in the organelle of epithelial tubule cells [2,32].

**Figure 2:**
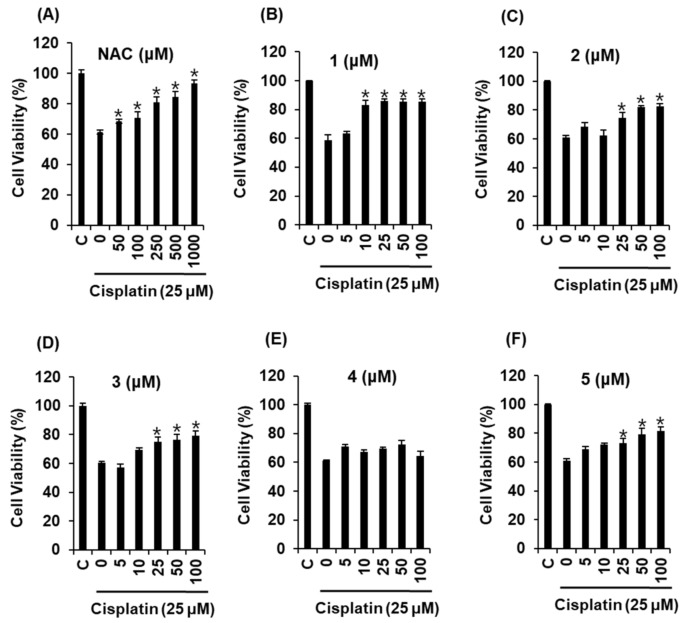
Comparison of protective effects of compounds **1**–**5** against cisplatin-induced cytotoxicity in LLC-PK1 kidney cell line. (**A**) Effect of positive control (*N*-acetylcysteine, NAC) on cisplatin-induced cytotoxicity; (**B**) Effect of compound **1**; (**C**) Effect of compound **2** on cisplatin-induced cytotoxicity; (**D**) Effect of compound **3** on cisplatin-induced cytotoxicity; (**E**) Effect of compound **4** on cisplatin-induced cytotoxicity; (**F**) Effect of compound **5** on cisplatin-induced cytotoxicity. * *p* < 0.05 compared to the cisplatin-treated value.

We tested the effects of the isolated compounds on cisplatin-induced kidney cell damage. The protective effect of compounds **1**–**5** against cisplatin-induced cytotoxicity in LLC-PK1 kidney cell line was evaluated in this study. This cell line is one of the most frequently used cell-based models for evaluating cisplatin-induced nephrotoxicity [33,34,35]. We performed comparative experiments with compounds **1**–**5**. The cytotoxicity of 25 μM cisplatin in LLC-PK1 cells was reversed by co-treatment with compounds **1**–**5** (Figure 2). The effect of compound **1** was the most pronounced among the five compounds.

**Figure 3:**
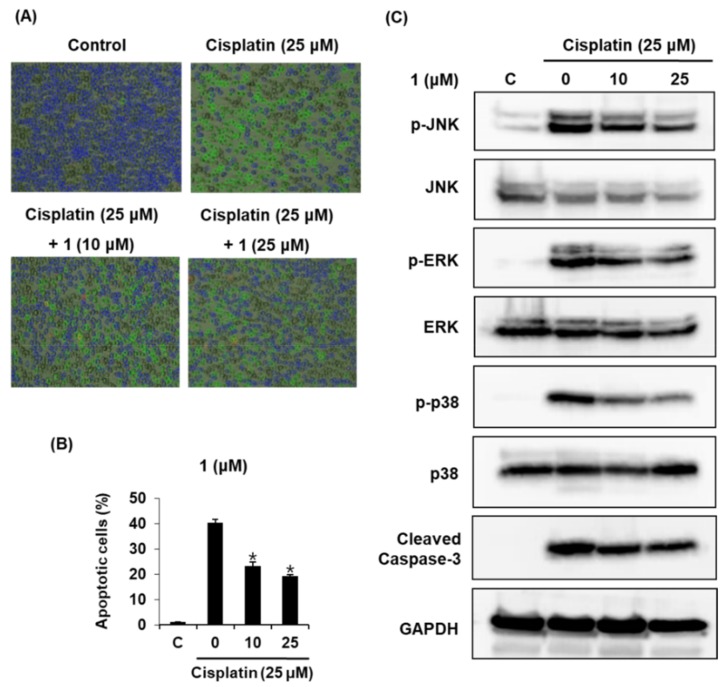
Mechanism of protective effect of compound **1** against cisplatin-induced apoptosis in LLC-PK1 kidney cells. (**A**) Representative images of apoptotic cells (magnifications of ×4); (**B**) Percentage of apoptotic cells; (**C**) Compound **1** activates the MAPK-caspase-3 pathway in LLC-PK1 kidney cell line. * *p* < 0.05 compared to the cisplatin-treated value.

*N*-Acetylcysteine (NAC) was used as a positive control protects against cisplatin-induced kidney cell death [36]. As shown in Figure 2, compared with the positive control, compound **1** exerted more than 83.14 ± 3.15% protective effects at 10 μM. The protective effect of compound **1** was quite stronger than that of NAC. We further explored whether compound **1** could decrease cisplatin-induced apoptosis in LLC-PK1 cells through annexin V Alexa Fluor 488 and propidium iodide staining. As shown in Figure 3A, apoptotic cell death (indicated by annexin V staining) was increased by 25 μM cisplatin from 1.0 ± 0.0% to 40.3 ± 1.5%, whereas it decreased after co-treatment with compound **1** (10, 25 μM) by 19.3 ± 0.57%. The percentage of apoptotic cells is shown in the bar graph in Figure 3B. Regarding the effect of compound **1** (10 and 25 μM) on mitogen-activated protein kinase (MAPK) protein expression in cisplatin-induced apoptosis in LLC-PK1 cells, the phosphorylation of Jun N-terminal kinase (JNK), extracellular signal-regulated kinase (ERK), p38, mitogen-activated protein kinase (MAPK), and cleaved caspase-3 decreased after treatment with compound **1** (10 and 25 μM) in a concentration-dependent manner (Figure 3C).

The cisplatin-induced nephrotoxicity occurs in combination with an interconnected pathway such as oxidative stress, inflammatory, MAPK, and apoptosis pathways [31,32]. MAPKs are crucial enzymes involved in various intracellular pathways, such as cell differentiation, proliferation, survival, and death. They are composed of JNK, p38, and ERK, which are detectable in various renal cells [37].

JNK and p38 are induced by cellular stress, inflammatory response, and apoptotic pathways, and activated in renal ischemia–reperfusion. ERK is mostly induced by cell death and survival growth factors and activated in toxic renal injury [31,38]. The apoptotic pathway in cisplatin-induced apoptosis is characterized by the loss of renal cells that provoke the dysfunction of the kidney. Cisplatin impairs the pathogenesis of nephrotoxicity through the expression of caspase-3, which plays an important role in the apoptotic pathway [39,40]. In line with these studies, our results demonstrated that the phosphorylation of JNK, ERK, p38 MAP kinase, and caspase-3 was increased by cisplatin, but decreased after treatment with compound **1**, and that the percentage of apoptotic cells also decreased.

## 3. Materials and Methods

### 3.1. General Experimental Procedures

Optical rotations were calculated using a Jasco P-1020 polarimeter (Jasco, Easton, MD, USA). Infra red spectra were recorded using a Bruker IFS-66/S FT-IR spectrometer (Bruker, Karlsruhe, Germany). Ultraviolet–visible (UV) spectra were obtained on an Agilent 8453 UV–visible spectrophotometer (Agilent Technologies, Milford, MA, USA). LC/MS analysis was conducted on an Agilent 1200 Series HPLC system equipped with a diode array detector and 6130 Series ESI mass spectrometer (Agilent Technologies). NMR spectra, including ^1^H–^1^H correlation spectroscopy, heteronuclear single-quantum correlation, and heteronuclear multiple bond correlation experiments, were documented using a Bruker AVANCE III 700 NMR spectrometer (Bruker, Madison, WI, USA) operating at 700 MHz (^1^H) and 175 MHz (^13^C), respectively. In preparative HPLC, we used Waters 1525 binary HPLC pump with Waters 996 photodiode array detector and Agilent Eclipse C18 column (21.2 × 250 mm; flow rate: 5 mL/min). In semi-preparative HPLC, Shimadzu Prominence HPLC System with SPD-20A/20AV Series Prominence HPLC UV–vis Detectors and Phenomenex Luna HPLC phenyl-hexyl column (250 × 10 mm; flow rate: 2 mL/min) was used. Column chromatography was performed with a silica gel 60 (Merck, Frankfurt, Germany, 230–400 mesh), RP-C18 silica gel (Merck, 230–400 mesh) and HP-20 dianion column. Merck precoated silica gel F254 plates and reversed-phase (RP)-18 F254s plates were utilized for thin-layer chromatography. Spots were detected on TLC under UV light or heated under anisaldehyde–sulfuric acid dye.

### 3.2. Plant Material

The fruits of *M. alba* were obtained from the Kyungdong Market (Woori Herb), Seoul, Korea, in January 2014. A voucher specimen (MA 1414) of the material was confirmed by one of the authors (Ki Hyun Kim) and located in laboratory 306 of the Dong-A ST Research Center, Yongin, Korea.

### 3.3. Extraction and Isolation

Dried and mashed *M. alba* fruits (9.7 kg) were extracted by mixing the material with 70% aqueous EtOH thrice at room temperature and filtered. After evaporation of the filtrate in vacuo, the resultant residue (1.4 kg) was dissolved in deionized water and solvent-partitioned with hexane, CH_2_Cl_2_, EtOAc, and *n*-BuOH (each of 800 mL × 3), which afforded 27.8, 85.3, 32.9, and 138.8 g of each fraction, respectively. The *n*-BuOH-soluble fraction (138.8 g) was subjected to a HP-20 dianion column and washed with H_2_O and MeOH to give two fractions: MeOH-soluble fraction (14.1 g) and water-soluble fraction (57.7 g). The MeOH fraction (14.1 g) was separated using a silica gel (230−400 mesh) column and CH_2_Cl_2_–MeOH–H_2_O (9:3:0.1) into five fractions (A–E). Fraction A (2.6 g) was subjected to an RP-C18 silica gel (230–400 mesh) column chromatography (elution with 40% MeOH) to obtain seven fractions (A1–A7). Fraction A6 (640 mg) was separated using preparative reversed-phase HPLC using Agilent Eclipse C18 column (21.2 × 250 mm; flow rate: 5 mL/min) and 30% MeOH into six subfractions (A61–66). Subfraction A65 (18 mg) was separated using semi-preparative reversed-phase HPLC using Phenomenex Luna HPLC phenyl-hexyl column (250 × 10 mm; flow rate: 2 mL/min) and 20% MeCN to obtain compound **1** (4.0 mg, *t*_R_ = 44.0 min). Fraction B (1.2 g) was subjected to an RP-C18 silica gel (230−400 mesh) column chromatography (elution with 10%, 30%, 50%, and 100% MeOH/H_2_O) to obtain four fractions (B1–B4). Fraction B3 (258 mg) was isolated by preparative reversed-phase HPLC using Agilent Eclipse C18 column (21.2 × 250 mm; flow rate: 5 mL/min) with 30% MeOH to obtain seven subfractions (B31–B37). Subfraction B32 (31.1 mg) was purified by semi-preparative reversed-phase HPLC using Phenomenex Luna HPLC phenyl-hexyl column (250 × 10 mm; flow rate: 2 mL/min) with 13% MeCN to furnish compound **5** (0.5 mg, *t*_R_ = 44.0 min). Subfraction B34 (18.3 mg) was purified by semi-preparative reversed-phase HPLC with 45% MeOH to yield compound **3** (5.7 mg, *t*_R_ = 40.0 min) using Phenomenex Luna HPLC phenyl-hexyl column (250 × 10 mm; flow rate: 2 mL/min). Fraction B35 (27.5 mg) was isolated with semi-preparative HPLC using Phenomenex Luna HPLC phenyl-hexyl column (250 × 10 mm; flow rate: 2 mL/min) with 30% MeOH to obtain compound **2** (5.7 mg, *t*_R_ = 34.0 min). Finally, fraction D (0.6 g) was subjected to an RP-C18 silica gel (230–400 mesh) column chromatography (elution with 50% MeOH) to obtain four subfractions (D1–D4). Subfraction D4 (80.3 mg) was also subjected to semi-preparative reversed-phase HPLC using Phenomenex Luna HPLC phenyl-hexyl column (250 × 10 mm; flow rate: 2 mL/min) with 18% MeCN to obtain compound **4** (7.0 mg, *t*_R_ = 21.5 min).

### 3.4. Cell Culture

LLC-PK1 kidney cell line was purchased from the American Type Culture Collection (Rockville, MD, USA); cultured in Dulbecco’s modified Eagle’s medium (DMEM) (Cellgro, Manassas, VA, USA) supplemented with 100 units/mL penicillin, 10% FBS, and 100 μg/mL streptomycin; and cultured at 37 °C in a humidified atmosphere with 5% CO_2_.

### 3.5. Renoprotective Effect against Cisplatin-Induced Cytotoxicity in LLC-PK1 Kidney Cells

The LLC-PK1 cells were seeded on 96-well culture plates at 1 × 10^4^ cells per well and incubated with different concentrations of compounds **1**–**5** and/or 25 μM cisplatin. After incubation for 24 h, the medium containing all the compounds was removed. Ez-Cytox reagent (10 µL) was added to each well and incubated at 37 °C for 1 h. The viability of the cells was measured using a microplate reader at an absorbance of 450 nm (PowerWave XS; Bio-Tek Instruments, Winooski, VT, USA).

### 3.6. Western Blot Analysis

Whole cell lysates for Western blot analysis were prepared in a lysis buffer (Cell Signaling, Danvers, MA, USA) containing 1 mM phenylmethylsulfonyl fluoride. The proteins (20 µg) were applied to electrophoresis in a pre-casted 4–15% Mini-PROTEAN TGX gel (Bio-Rad, Hercules, CA, USA) and blotted onto PVDF transfer membranes. Then, the membranes were blocked in 5% skim milk at room temperature for 1 h and analyzed with epitope-specific primary and secondary antibodies. Each antibody was detected and visualized by ECL Advance Western Blotting Detection Reagents (GE Healthcare, Amersham, UK) and a FUSION Solo Chemiluminescence System (PEQLAB Biotechnologie GmbH, Erlangen, Germany).

### 3.7. Statistical Analysis

The data are presented as the mean standard deviation (SD). Statistical significance was determined using Mann–Whitney U test. *p*-Values < 0.05 were considered statistically significant. 

## 4. Conclusions

In the present study, we identified five compounds in mulberry fruits. The isolated compounds **1**–**5** were evaluated for their effects on anticancer drug-induced side effects by using cell-based assays. Of the identified compounds in the mulberry fruits, compound **1**, butyl pyroglutamate, showed the most pronounced protective effect against cisplatin-induced kidney cell damage. This effect was found to be mediated by the attenuation of phosphorylation of JNK, ERK, p38 MAP kinase, and caspase-3 in cisplatin-induced kidney cell damage. The results of our study may provide basis for the development of novel protective agents that can be used in the treatment of cisplatin-induced nephrotoxicity.

## References

[1] Ma P., Zhang S., Su X., Qiu G., Wu Z. (2015). Protective effects of icariin on cisplatin-induced acute renal injury in mice. Am. J. Transl. Res..

[2] Peres L.A.B., Cunha Júnior A.D. (2013). Acute nephrotoxicity of cisplatin: Molecular mechanisms. J. Bras. Nefrol..

[3] Yin J., Wang J. (2016). Renal drug transporters and their significance in drug—Drug interactions. Acta Pharm. Sin. B.

[4] Havasi A., Borkan S.C. (2011). Apoptosis and acute kidney injury. Kidney Int..

[5] Yao X., Panichpisal K., Kurtzman N., Nugent K. (2007). Cisplatin nephrotoxicity: A review. Am. J. Med. Sci..

[6] Mohrmann M., Ansorge S., Schmich U., Schönfeld B., Brandis M. (1994). Toxicity of ifosfamide, cyclophosphamide and their metabolites in renal tubular cells in culture. Pediatr. Nephrol..

[7] Servais H., Ortiz A., Devuyst O., Denamur S., Tulkens P.M., Mingeot-Leclercq M.P. (2008). Renal cell apoptosis induced by nephrotoxic drugs: Cellular and molecular mechanisms and potential approaches to modulation. Apoptosis.

[8] Ries F., Klastersky J. (1986). Nephrotoxicity induced by cancer chemotherapy with special emphasis on cisplatin toxicity. Am. J. Kidney Dis..

[9] Kintzel P.E. (2001). Anticancer drug—Induced kidney disorders. Drug Saf..

[10] Leekha A., Gurjar B.S., Tyagi A., Rizvi M.A., Verma A.K. (2016). Vitamin C in synergism with cisplatin induces cell death in cervical cancer cells through altered redox cycling and p53 upregulation. J. Cancer Res. Clin. Oncol..

[11] Zhen S., Lu J.J., Wang L.J., Sun X.M., Zhang J.Q., Li X., Luo W.J., Zhao L. (2016). In vitro and in vivo synergistic therapeutic effect of cisplatin with human papillomavirus16 E6/E7 CRISPR/Cas9 on cervical cancer cell line. Transl. Oncol..

[12] McKim A., Walter A., Sheely K., Manahan K., Geisler J. (2016). An economic analysis of cisplatin alone versus cisplatin doublets in the treatment of women with advanced or recurrent cervical cancer. Eur. J. Gynaecol. Oncol..

[13] Wang S., Xie J., Li J., Liu F., Wu X., Wang Z. (2016). Cisplatin suppresses the growth and proliferation of breast and cervical cancer cell lines by inhibiting integrin β5-mediated glycolysis. Am. J. Cancer Res..

[14] Han M.S., Han I.H., Lee D., An J.M., Kim S.N., Shin M.S., Yamabe N., Hwang G.S., Yoo H.H., Choi S.J. (2016). Beneficial effects of fermented black ginseng and its ginsenoside 20 (S)-Rg3 against cisplatin-induced nephrotoxicity in LLC-PK1 cells. J. Ginseng Res..

[15] Kim N., Min W.K., Park M.H., Lee J.K., Jin H.K., Bae J.S. (2016). Neuropeptide Y protects kidney against cisplatin-induced nephrotoxicity by regulating p53-dependent apoptosis pathway. BMB Rep..

[16] Huang H.P., Ou T.T., Wang C.J. (2013). Mulberry (sang shen zi) and its bioactive compounds, the chemoprevention effects and molecular mechanisms in vitro and in vivo. J. Tradit. Complement. Med..

[17] Kim Y.R., Lee J.S., Lee K.R., Kim Y.E., Baek N.I., Hong E.K. (2014). Effects of mulberry ethanol extracts on hydrogen peroxide-induced oxidative stress in pancreatic β-cells. Int. J. Mol. Med..

[18] Salih N.D., Hazir N.S.M., Hamid M.H.A. (2015). The effect of mulberry (*Morus* sp.) tea supplement on acetaminophen induced renal failure in rats. Lab. Sci..

[19] Peanparkdee M., Iwamoto S., Borompichaichartkul C., Duangmal K., Yamauchi R. (2016). Microencapsulation of bioactive compounds from mulberry (*Morus alba* L.) leaf extracts by protein–polysaccharide Interactions. Int. J. Food Sci. Technol..

[20] Grajek K., Wawro A., Kokocha D. (2015). Bioactivity of *Morus alba* L. extracts—An overview. Int. J. Pharm. Sci. Res..

[21] Memon A.A., Memon N., Luthria D.L., Bhanger M.I., Pitafi A.A. (2010). Phenolic acids profiling and antioxidant potential of mulberry (*Morus laevigata* W., *Morus nigra* L., *Morus alba* L.) leaves and fruits grown in Pakistan. Pol. J. Food Nutr. Sci..

[22] Andallu B., Suryakantham V., Lakshmi Srikanthi B., Reddy G.K. (2001). Effect of mulberry (*Morus indica* L.) therapy on plasma and erythrocyte membrane lipids in patients with type 2 diabetes. Clin. Chim. Acta.

[23] Lee S.R., Park J.Y., Yu J.S., Lee S.O., Ryu J.Y., Choi S.Z., Kang K.S., Yamabe N., Kim K.H. (2016). Odisolane, a novel oxolane derivative, and antiangiogenic constituents from the fruits of mulberry (*Morus alba* L.). J. Agric. Food Chem..

[24] Yuping T., Biao Y., Jie H., Tao W., Yongzheng H. (2001). The chemical constituents from the bulbs of Ornithogalum caudatum. J. Chin. Pharm. Sci..

[25] Han J.T., Bang M.H., Chun O.K., Kim D.O., Lee C.Y., Baek N.I. (2004). Flavonol glycosides from the aerial parts of *Aceriphyllum rossii* and their antioxidant activities. Arch. Pharm. Res..

[26] Sang S.M., Lapsley K., Jeong W.S., Lachance P.A., Ho C.T., Rosen R.T. (2002). Antioxidative phenolic compounds isolated from almond skins (*Prunus amygdalus* batsch). J. Agric. Food Chem..

[27] Li Y.L., Li J., Wang N.L., Yao X.S. (2008). Flavonoids and a new polyacetylene from *Bidens parviflora* Willd. Molecules.

[28] Baumes R., Bayonove C., M’Bairaroua O., Tapiero C. (1990). Synthesis and NMR spectral properties of grape monoterpenyl glycosides. Carbohydr. Res..

[29] Florea A.-M., Büsselberg D. (2011). Cisplatin as an anti-tumor drug: Cellular mechanisms of activity, drug resistance and induced side effects. Cancers.

[30] Rajeswaran A., Trojan A., Burnand B., Giannelli M. (2008). Efficacy and side effects of cisplatin-and carboplatin-based doublet chemotherapeutic regimens versus non-platinum-based doublet chemotherapeutic regimens as first line treatment of metastatic non-small cell lung carcinoma: A systematic review of randomized controlled trials. Lung Cancer.

[31] Lee H.L., Kang K.S. (2017). Protective effect of ginsenoside Rh3 against anticancer drug-induced apoptosis in LLC-PK1 kidney cells. J. Ginseng Res..

[32] Rodriguez-Garcia M.E., Quiroga A.G., Castro J., Ortiz A., Aller P., Mata F. (2009). Inhibition of p38-MAPK potentiates cisplatin-induced apoptosis via GSH depletion and increases intracellular drug accumulation in growth-arrested kidney tubular epithelial cells. Toxicol. Sci..

[33] Lee D., Kim K.H., Moon S.W., Lee H., Kang K.S., Lee J.W. (2015). Synthesis and biological evaluation of chalcone analogues as protective agents against cisplatin-induced cytotoxicity in kidney cells. Bioorg. Med. Chem. Lett..

[34] Kim T., Kim Y.J., Han I.H., Lee D., Ham J., Kang K.S., Lee J.W. (2015). The synthesis of sulforaphane analogues and their protection effect against cisplatin induced cytotoxicity in kidney cells. Bioorg. Med. Chem. Lett..

[35] Kang H.R., Lee D., Eom H.J., Lee S.R., Lee K.R., Kang K.S., Kim K.H. (2016). Identification and mechanism of action of renoprotective constituents from peat moss Sphagnum palustre in cisplatin-induced nephrotoxicity. J. Funct. Foods.

[36] Abdelrahman A.M., Al Salam S., AlMahruqi A.S., Mansour M.A., Ali B.H. (2010). *N*-acetylcysteine improves renal hemodynamics in rats with cisplatin-induced nephrotoxicity. J. Appl. Toxicol..

[37] Tian W., Zhang Z., Cohen D.M. (2000). MAPK signaling and the kidney. Am. J. Physiol. Ren. Physiol..

[38] Omori S., Hida M., Ishikura K., Kuramochi S., Awazu M. (2000). Expression of mitogen-activated protein kinase family in rat renal development. Kidney Int..

[39] Yang B., Johnson T.S., Thomas G.L., Watson P.F., Wagner B., Nahas A.M. (2001). Apoptosis and caspase-3 in experimental anti-glomerular basement membrane nephritis. J. Am. Soc. Nephrol..

[40] Jeon J.H., Kim D.K., Shin Y., Kim H.Y., Song B., Lee E.Y., Kim J.K., You H.J., Cheong H., Shin D.H. (2016). Migration and invasion of drug-resistant lung adenocarcinoma cells are dependent on mitochondrial activity. Exp. Mol. Med..

